# Monitoring and Biosurveillance Tools for the Brown Marmorated Stink Bug, *Halyomorpha halys* (Stål) (Hemiptera: Pentatomidae)

**DOI:** 10.3390/insects9030082

**Published:** 2018-07-08

**Authors:** Angelita L. Acebes-Doria, William R. Morrison, Brent D. Short, Kevin B. Rice, Hayley G. Bush, Thomas P. Kuhar, Catherine Duthie, Tracy C. Leskey

**Affiliations:** 1 Department of Entomology, University of Georgia, Tifton, GA 31793, USA; 2 USDA-ARS, Center for Grain and Animal Health Research, Manhattan, KS 66502, USA; william.morrison@ars.usda.gov; 3 USDA-ARS, Appalachian Fruit Research Station, Kearneysville, WV 25430, USA; brent.short@ars.usda.gov (B.D.S.); tracy.leskey@ars.usda.gov (T.C.L.); 4 Division of Plant Sciences, University of Missouri, Columbia, MO 65201, USA; ricekev@missouri.edu; 5 Department of Entomology, Virginia Tech, Blacksburg, VA 24060, USA; hgbush93@vt.edu (H.G.B.); tkuhar@vt.edu (T.P.K.); 6 Ministry for Primary Industries, Wellington 6140, New Zealand; Catherine.Duthie@mpi.govt.nz

**Keywords:** trapping, lure comparison, invasive species, pest management

## Abstract

*Halyomorpha halys* (Stål) (Hemiptera: Pentatomidae) is an invasive pest of numerous agricultural crops with an increasing global distribution. Finding simple and reliable monitoring tools for *H. halys* agricultural and surveillance programs is imperative. In 2016, we compared standard pyramid traps to clear sticky cards attached atop wooden stakes and evaluated two commercially formulated lures (Trécé and AgBio) with low and high rates of the *H. halys* aggregation pheromone (PHER) and pheromone synergist (MDT) at 12 sites (low: 5 mg PHER + 50 mg MDT; high: 20 mg PHER + 200 mg MDT). In 2017, we reevaluated lure efficacy using only the clear sticky traps at six locations. Sites were classified as having low, moderate, or high relative population densities of *H. halys* in 2016, and as very low or low densities of *H. halys* in 2017. Although clear sticky traps captured fewer adults and nymphs than pyramid traps, their captures were generally correlated at all population levels indicating that clear sticky traps can reliably monitor *H. halys* presence and relative abundance regardless of relative population density. During both years, adult and nymphal captures were significantly greater in traps baited with Trécé lures than with AgBio lures. Captures were greater in traps baited with high loading rate lures for each lure type, and with the exception of traps baited with AgBio lures at high relative density sites in 2016, *H. halys* captures in traps with low and high loading rates of each lure type were correlated for both years. Comparison of yellow and clear sticky cards indicated they performed equally, but yellow cards captured more nontargets. In summary, clear sticky traps attached atop wooden posts and baited with *H. halys* pheromone and pheromone synergist lures are an effective option for this pest monitoring and detection.

## 1. Introduction

Monitoring pest populations is critical in agricultural systems and for biosurveillance of invasive pests at high-risk areas and newly invaded locations. In agriculture, treatment thresholds based on monitoring tools can be used to effectively manage pest populations, while reducing production costs and nontarget effects [1]. In biosurveillance programs, effective monitoring and detection tools can provide accurate information on the presence, establishment and distribution of invasive pests in new areas, thereby ensuring the potential for timely action for eradication and/or reducing the rate of spread [2]. The need to develop an effective and reliable monitoring tool for the purpose of pest management and biosurveillance is particularly pertinent to the brown marmorated stink bug, *Halyomorpha halys* (Stål) (Hemiptera: Pentatomidae).

*Halyomorpha halys* is an invasive species from Asia that has become one of the most economically important pests for numerous agricultural crops including tree fruit, row crops, vegetables, and ornamentals [3,4]. An estimated $37M USD losses in apple production was attributed to this pest in the US mid-Atlantic in 2010 [3]. Currently, established populations have been found in many US states and other countries including Canada [5], Switzerland [6], Italy [7], Republic of Georgia [8], Romania [9] and Chile [10]. Due to its increasing distribution and potential risk of establishment in other areas [11,12] which can be partly attributed to human-mediated dispersal (hitchhiking), countries like New Zealand have initiated development of biosurveillance programs to detect presence of *H. halys* before they become well-established [13]. Hence, the need to find an effective, practical and user-friendly trap for *H. halys* monitoring either under the framework of integrated pest management and/or biosurveillance has become paramount.

In recent years, there have been several studies evaluating various factors affecting trap effectiveness for monitoring *H. halys* including trap designs, colors, pheromone lures, deployment strategies, and capture mechanisms [14,15,16,17,18,19]. Based on these studies, the black pyramid trap made of corrugated plastic has become the standard trap and standard lures consist of the aggregation pheromone (3*S*,6*S*,7*R*,10*S*)-10,11-epoxy-1-bisabolen-3-ol and (3*R*,6*S*,7*R*,10*S*)-10,11-epoxy-1-bisabolen-3-ol [20] in combination with methyl (2*E*,4*E*,6*Z*)-decatrienoate (MDT), a pheromone synergist [21,22]. This trap design and lure combination has effectively monitored adult and nymphal *H. halys* populations season long across the USA [22] and provided efficacious decision support tools for pest management decisions in apple orchards [23]. However, this trap is time-consuming to install, its size makes it cumbersome to handle and interferes with farm activities (A.L.A, B.D.S., personal communication). A recent study conducted by Rice et al. [18] showed that *H. halys* captures using simpler and smaller trap designs such as delta traps and yellow sticky traps hung in apple trees were positively correlated with captures in black pyramid traps with the degree of correlation being higher for yellow sticky traps. In Asia, pheromone-baited yellow sticky cards have also been used for trapping the Oriental stink bug, *Plautia stali* Scott (Hemiptera: Pentatomidae) [24]. Although, the use of yellow sticky cards showed promise for *H. halys* trapping, using these cards imposes a risk of capturing numerous nontargets as yellow offers a super-normal visual stimulus to numerous insects [25]. This emphasizes the importance of developing a more target-specific sticky trap design for *H. halys*. Moreover, apart from the trap design itself, there is also the need to identify effective lure rates and formulations that are manufactured commercially which can be made available for use to the public.

Hence, the main objective of this study was to further refine tools for monitoring *H. halys* considering lure efficacy, trap effectiveness, simplicity and target specificity. In 2016, we compared the effectiveness of the standard pyramid trap with a clear plastic sticky card deployed on a wooden stake, and baited with lures at low and high loading rates from two different manufacturers. We also compared yellow sticky cards to the other trap designs to evaluate capture rates of both *H. halys* and nontargets. In 2017, we evaluated the efficacy of the two commercial lures at low and high loading rates once again using the clear sticky traps.

## 2. Materials and Methods 

### 2.1. 2016 Trap and Lure Comparisons

**Figure 1:**
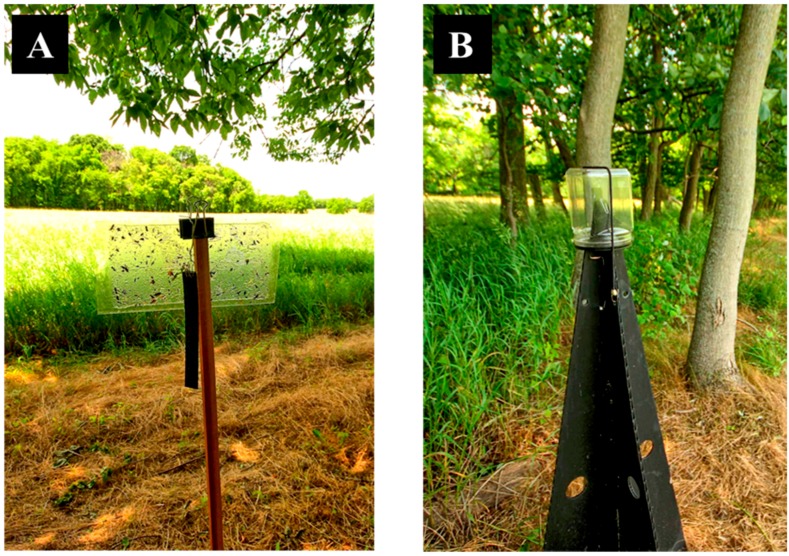
The two traps compared during the 2016 and 2017 studies: (**A**) standard black pyramid trap and (**B**) double-sided clear sticky card clipped to the top of a wooden stake. Both traps were baited with commercially formulated *H. halys* lures.

In 2016, we compared *H. halys* captures between standard pyramid traps [17] and clear sticky traps deployed along the perimeter of agricultural areas adjacent to woods across 12 sites in West Virginia (WV) and Virginia (VA), USA (Table 1). Traps at these sites were arrayed in four replicate transects, with eight traps spaced 50 m apart in each transect. Four of the traps were large, black Coroplast Dead-Inn pyramid traps (Figure 1A, 1.2 m height, AgBio Inc., Westminster, CO, USA) while the other four were double-sided clear sticky cards (Figure 1B, 15.2 × 30.5 cm, Trécé, Inc., Adair, OK, USA). We alternated the trap placement in the field within each transect every week. Atop each pyramid trap was a clear collection jar containing deltamethrin-impregnated netting (Vestergaard-Frandsen Inc., Lausanne, Switzerland) held to the entire surface of the interior funnel by a paper clip [18]. Clear sticky traps were attached horizontally by black steel binder clips (5 cm, Skilcraft LLC, Burlington, KY, USA) and stapled in the bottom to the top of 1.5-m wooden stakes (2.5 cm^2^, redwood), with 0.30 m of the stake buried in the soil. At select sites in WV (Table 1), we also deployed double-sided yellow sticky traps (20.3 × 27.9 cm, Alpha Scents, West Linn, OR, USA) horizontally attached atop wooden stakes to assess their efficacy relative to the other trap types. Four lure treatments were evaluated and included low loading and high loading rate lures manufactured by AgBio and Trécé. Low loading consisted of 5 mg of the *H. halys* aggregation pheromone and 50 mg of the methyl (2*E*,4*E*,6*Z*)-decatrienoate (MDT) pheromone synergist (Trécé low and AgBio low, hereafter), while the high loading rates contained 20 mg of the *H. halys* aggregation pheromone and 200 mg of MDT (Trécé high and AgBio high, hereafter). Lures were secured by metal wires outside the collection jars of pyramid traps as this was found to increase trap captures compared with lures deployed inside collection jars [18] and attached to the binder clip when paired with sticky cards to prevent contact with the glue (Figure 1B). Lure treatments were randomized in each transect. At the Virginia sites, the AgBio lures were not deployed until the week of 1 August 2016 (Eastern Shore) and 27 June 2016 (Goochland 1, 2 and 3). AgBio lures were replaced every four weeks, while Trécé lures were replaced every eight weeks based on a previously established protocol (B.D.S., unpublished). Each trap was checked weekly for the presence of *H. halys* adults and nymphs from mid-May to early October 2016 (see exact dates on Table 1). At select sites in WV with both yellow and clear sticky cards, the percent coverage of the sticky cards was visually estimated to account for nontarget captures. Sticky cards were replaced every two weeks, and all lure treatments were rerandomized every four weeks within each replicate transect during the growing season.

### 2.2. 2017 Lure Comparison using Clear Sticky Traps

In 2017, we repeated the experiment comparing the same two commercially formulated lures, Trécé and AgBio, at low and high loadings with double-sided clear sticky cards at six sites in Virginia and West Virginia (Table 1). We only used clear sticky traps in 2017 to further investigate its effectiveness in combination with the different lure formulations. Following the 2016 protocol, traps at these sites were arranged in four replicate transects, with four traps spaced 50 m apart in each transect. The same protocol from the previous year for lure randomization and replacement was followed. The number of adult and nymphal *H. halys* captures was counted weekly from mid-May to mid-October 2017 (see exact dates on Table 1). Sticky cards were replaced every two weeks or if ≥50% of the card was covered with insects or debris on either side. 

### 2.3. Statistical Analyses

**Figure 2:**
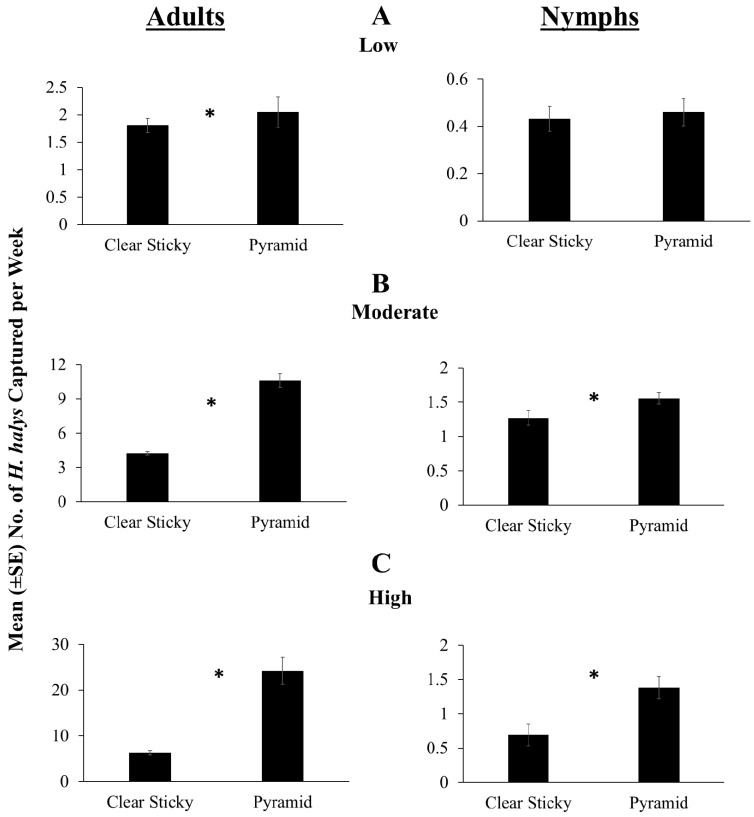
Results comparing the effectiveness of clear sticky traps and pyramid traps under (**A**) low, (**B**) moderate and (**C**) high *H. halys* relative population densities. Data pooled across all lure treatments. Due to the differences in the sampling period at the different locations, data obtained from 31 May to 3 October were used for the low relative population density sites while for moderate and high relative population density sites data were analyzed from 23 May to 3 October 2016. Asterisk indicates significant difference between the two trap designs at α = 0.05.

**Figure 3:**
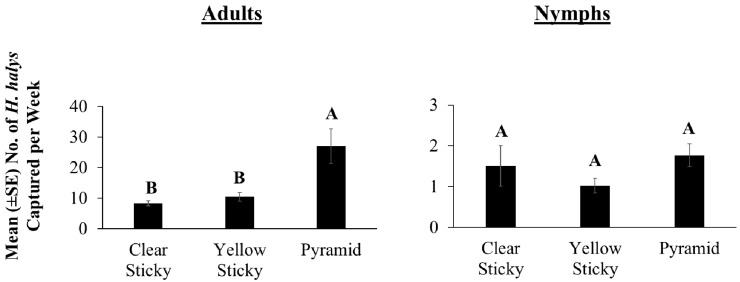
Comparison of *H. halys* adult and nymphal captures among three trap designs baited with Trécé low loading lures. Captures in these traps were compared across three sites in West Virginia from 26 June to 3 October 2016. Bars with shared letters are not significantly different from each other at α = 0.05 (*χ*^2^ mean contrast with Bonferroni correction).

In both 2016 and 2017, we pooled the adult capture data across all treatments to analyze the differences in population densities among field sites using generalized linear regression model (GLM) with zero-inflated (ZI) Poisson distribution because of an overabundance of zero observations [26]. Subsequently, we used a multiple mean comparison procedure designed for generalized linear models based on *χ*^2^ statistics, with a Bonferroni correction, and the resulting post-hoc results were used to classify the relative population density (Figure 2 and Figure 3). To ensure that the mean adult captures in each population group are statistically distinct, we employed the same GLM ZI Poisson model and post-hoc analysis in analyzing mean adult captures at all sites to designate sites as having very low, low, moderate or high relative densities of *H. halys*. We then analyzed treatment effects separately for high, moderate, and low relative density sites in 2016, and for low and very low relative density sites in 2017. To examine differences of nymphal and adult captures among treatments for each site in each year (2016 treatments: lure type and trap design, 2017 treatment: lure type), we used the same procedure as above. In each analysis, we only analyzed data during sampling periods when all treatments were represented. A *t*-test was conducted to compare the percent coverage of the yellow and clear sticky cards deployed at three sites in WV from 26 June to 3 October 2016. Pearson correlations were run between the captures in pyramid traps and clear sticky cards baited with high and low rates of Trécé and AgBio lures in 2016. Pearson correlation analysis was also conducted to examine the relationship between captures in low and high rates of Trécé and AgBio lures on clear sticky cards in 2016 and 2017. Data subjected to *t*-test and Pearson correlation analyses were log-transformed to satisfy assumptions of parametric tests. We added “1”to the values prior to the log-transformation. All statistical analyses were conducted using JMP^®^ Pro, Version 13 (SAS Institute Inc., Cary, NC, USA).

## 3. Results

### 3.1. Relative Population Density Classifications

In 2016, three relative population densities were designated (low, moderate and high) across 12 field sites (Supplemental Material Figure S1; GLM ZI Poisson: *χ*^2^ = 11,651.43, *df* = 11, *p* < 0.0001). Mean weekly captures of *H. halys* adults was found to be significantly different with 1.93 ± 0.16, 7.2 ± 0.33 and 15.43 ± 1.53 adults/trap/week for low, moderate and high relative population density sites, respectively (GLM ZI Poisson: *χ*^2^ = 9758.31, *df* = 2, *p* < 0.0001). In 2017, very low and low relative population density groups were identified among the six sites (Supplemental Material Figure S2; GLM ZI Poisson: *χ*^2^ = 349.30, *df* = 5, *p* < 0.0001). Numbers of *H. halys* adults captured were significantly lower in low relative density sites compared with high relative density sites with 0.88 ± 0.06 and 2.60 ± 0.16 adults/trap/week, respectively (GLM ZI Poisson: *χ*^2^ = 291.63, *df* = 1, *p* < 0.0001).

### 3.2. 2016 Trap Design Comparison 

In low relative density sites, pyramid traps performed better than clear sticky cards in capturing more *H. halys* adults (Figure 2A; *χ*^2^ = 129.86, *df* = 1, *p* < 0.0001), while both traps performed equally well in capturing *H. halys* nymphs (Figure 2A; *χ*^2^ = 0.34, *df* = 1, *p* = 0.56). At moderate relative density sites, pyramid traps captured greater numbers of *H. halys* nymphs and adults than clear sticky cards (Figure 2B; adults: *χ*^2^ = 7666.42, *df* = 1, *p* < 0.0001; nymphs: *χ*^2^ = 9.48, *df* = 1, *p* = 0.0021), with pyramid traps capturing 1–2 times more nymphs and adults, respectively. At high relative density sites, pyramid traps captured significantly more adults and nymphs than clear sticky cards (Figure 2C; adults: *χ*^2^ = 8531.25, *df* = 1, *p* < 0.0001; nymphs: *χ*^2^ = 9.26, *df* = 1, *p =* 0.0023). In particular, pyramid traps captured 2–4 times more nymphs and adults, respectively (Figure 2C).

Significantly more *H. halys* adults were captured in pyramid traps than on clear or yellow sticky traps, while captures in both yellow and clear sticky trap were statistically equal to one another (Figure 3; *χ*^2^ = 3106.46, *df* = 2, *p* < 0.0001). On average, there were three times more adults captured in pyramid traps than on either kind of sticky trap. By contrast, there were no significant differences in nymphal captures among the three trap designs (Figure 3; *χ*^2^ = 43.20, *df* = 2, *p =* 0.34). Clear sticky cards had significantly fewer captures of nontarget insects than yellow sticky cards with 15% and 42% of the card area covered with nontargets, respectively (*t* = 14.71, *df* = 306, *p* < 0.0001).

**Figure 4:**
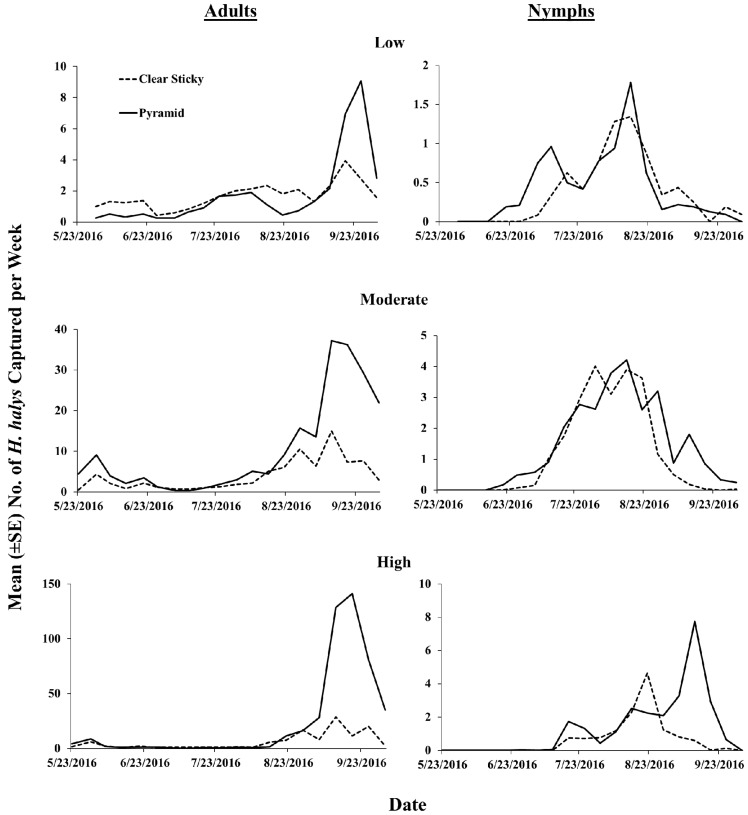
Seasonal trends in the captures of *H. halys* nymphs and adults using clear sticky traps and pyramid traps at low, moderate and high relative population density sites in 2016. Data pooled across all lure types and rates.

Based on the season-long captures at low, moderate and high relative population density sites pooled across different lure types, *H. halys* adult captures were greatest in the late season, with greater captures in pyramid traps compared with sticky cards at moderate and high relative population density sites (Figure 4). Nymphal captures generally peaked during the mid-season (Figure 4).

There were significant correlations between captures in the pyramid and clear sticky traps over the season. Based on the data pooled across all lure treatments, captures between pyramid and clear sticky traps for adults (Pearson correlation: *r* = 0.792, *df =* 37, *p* < 0.0001) and nymphs (*r* = 0.854, *df* = 37, *p* < 0.0001) were significantly correlated at low relative density sites. The same significant positive correlation held under moderate relative density sites for captures of adults (*r* = 0.666, *df* = 152, *p* < 0.0001) and nymphs (*r* = 0.553, *df* = 152, *p* < 0.0001) between pyramid and clear sticky traps. At high relative density sites, the same significant relationships were found for captures of adults (*r* = 0.747, *df* = 40, *p* < 0.0001) and nymphs (*r* = 0.360, *df* = 40, *p* = 0.02) between the pyramid and clear sticky traps. Using captures from traps baited with low and high loading rates of Trécé lures, we found strong significant positive correlations between the adult and nymphal captures in pyramid traps and those on clear sticky cards regardless of relative population density and *H. halys* life stage (Table 2). For traps baited with AgBio lures with high and low loading rates, we also found positive correlations between adult and nymphal captures in pyramid traps and those on clear sticky cards at all relative population densities, with the exception of the nymphal captures at high relative density sites.

### 3.3. 2016 Lure Comparison 

**Figure 5:**
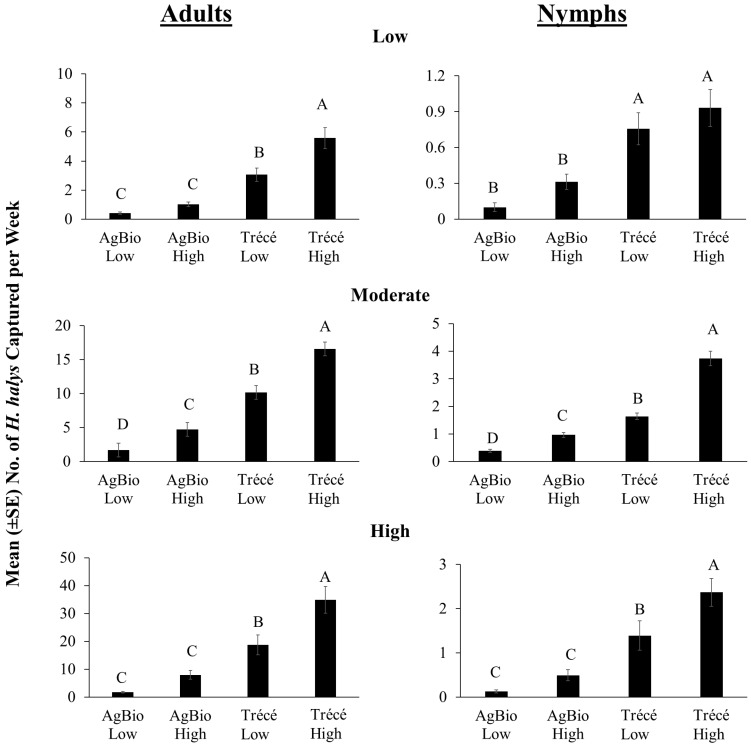
Mean weekly capture of *H. halys* using different lure types under low, moderate, or high relative population densities across 12 sites in the eastern United States in 2016. Data pooled among pyramid and clear sticky traps. Data in low, moderate and high relative population density sites were obtained from 1 August to 3 October, 27 June to 3 October and 6 June to 3 October 2016, respectively, ensuring all treatments were represented. Bars with shared letters are not significantly different from each other at α = 0.05 (*χ*^2^ mean contrast with Bonferroni correction).

**Figure 6:**
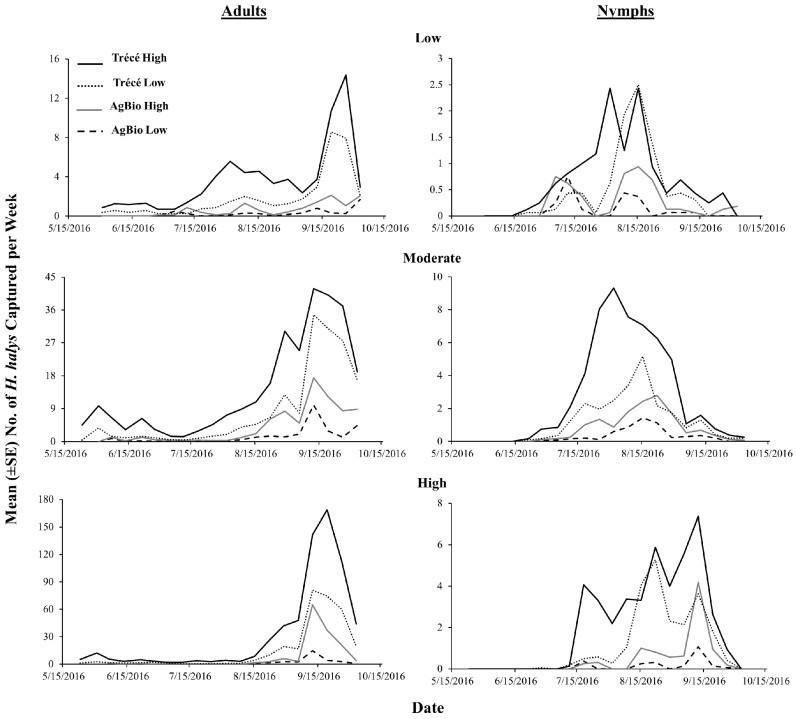
Seasonal trends in the captures of *H. halys* at low, moderate and high relative population density sites in the eastern United States in 2016 using four commercially formulated lures. Data pooled across pyramid and clear sticky traps.

Across trap designs, the type of lure significantly affected the captures of *H. halys* adults in traps at low, moderate, and high relative density sites (Figure 5: adults; low: *χ*^2^ = 330.06, *df* = 3, *p* < 0.0001; moderate: *χ*^2^ = 4028.40, *df* = 3, *p* < 0.0001; high: *χ*^2^ = 2786.66, *df* = 3, *p* < 0.0001). Consistently, traps baited with Trécé high loading lure captured significantly greater numbers of adults. Through the season, traps baited with Trécé high loading lure captured the greatest number of adults at low, moderate, and high relative population density sites (Figure 6: adults). Lure type also significantly affected trap captures of *H. halys* nymphs at low, moderate and high relative population density sites (Figure 5: nymphs; low: *χ*^2^ = 50.67, *df* = 3, *p* < 0.0001; moderate; *χ*^2^ = 771.73, *df* = 3; *p* < 0.0001; high: *χ*^2^ = 142.25, *df* = 3; *p* < 0.0001). Regardless of population, a similar pattern emerged with captures of nymphs in traps with high loading lures capturing the greatest numbers. With a few exceptions, traps baited with the Trécé high load lure captured the greatest number of nymphs at low, moderate and high relative population density sites (Figure 6: nymphs). When comparing captures on clear sticky cards with high and low rates of the Trécé lures, we found significant positive correlations in captures for both adults and nymphs, regardless of the relative population density (Table 3: 2016). The same was observed for AgBio lures except at high population density where captures on sticky traps baited with low loading lures were not significantly correlated with captures on sticky traps baited with high loading rate lures (Table 3: 2016). 

### 3.4. 2017 Lure Comparisons with Sticky Traps

**Figure 7:**
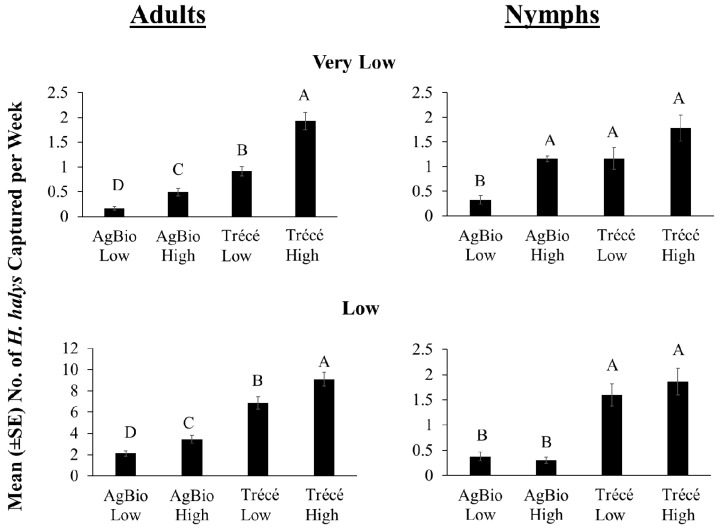
Mean weekly capture of *H. halys* using different lure types under very low and low relative population densities across six sites in the eastern United States in 2017. Due to differences in the trapping period among the sites, only dates that were common among the sites within each population density were included in the analyses. Data in low and high population sites were obtained from 5 June to 4 September and 22 May to 16 October 2017, respectively. Bars with shared letters are not significantly different from each other at α = 0.05 (*χ*^2^ mean contrast with Bonferroni correction).

**Figure 8:**
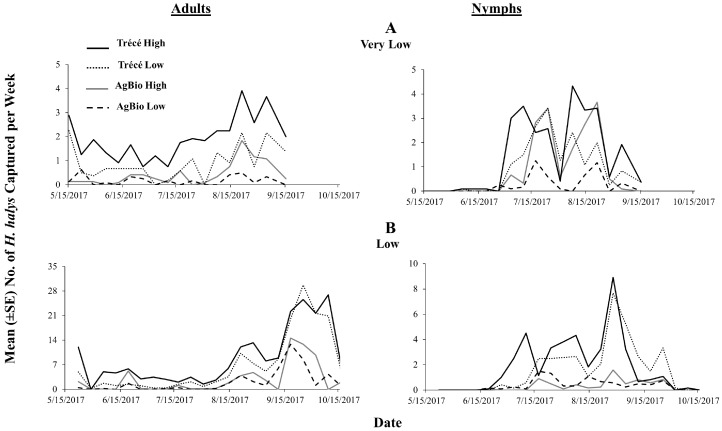
Seasonal trends in the captures of *H. halys* at (**A**) very low and (**B**) low relative population density sites in eastern United States in 2017 using four commercially formulated lures. Data pooled across pyramid and clear sticky traps.

At very low and low relative population density sites, captures of *H. halys* adults in baited clear sticky traps were significantly different among the four lure treatments, with traps baited with Trécé high loading lures capturing the greatest numbers (Figure 7: adults; low: *χ*^2^ = 166.05, *df* = 3, *p* < 0.0001; high: *χ*^2^ = 305.34, *df* = 3, *p* < 0.0001). Throughout the season, traps baited with Trécé high loading lure consistently captured greater numbers of adults compared with traps baited with other lures at very low relative population density sites (Figure 8A) and except for a single date, the same was observed at low relative population density sites (Figure 8B). For nymphal captures at very low relative population density sites, traps baited with AgBio low loading lures captured the least number of nymphs compared with traps baited with other lures, with the others being statistically equal (Figure 7: nymphs; *χ*^2^ = 20.95, *df* = 3, *p* < 0.0001). At low relative population density sites, nymphal captures did not differ significantly between traps baited with high and low loading rates within each manufacturer (Figure 7: nymphs; *χ*^2^ = 105.11, *df* = 3, *p* < 0.0001). Similar to the 2016 season-long trends, traps baited with the Trécé high loading lures captured the greatest number of nymphs through most of the season regardless of the relative population densities (Figure 8: nymphs). In 2017, we also found significant positive relationships of adult and nymphal captures on sticky traps baited with low and high loading rates of Trécé and with low and high loading rates of AgBio lures at very low and low population densities (Table 3: 2017).

## 4. Discussion

In commercial settings, the black pyramid trap has become the standard *H. halys* monitoring tool and a trap-based treatment threshold in apples has already been established using this trap type [23]; however, its size and labor requirement for installation make it less practical and less adoptable for grower use than smaller trap designs [18]. Hence, there has been the need to develop an easy-to-use and simple trap design for season-long monitoring of *H. halys* on commercial farms. The same simplified trapping approach may also be useful in the surveillance of *H. halys* in at-risk locations and in newly-invaded areas. Although there have been recent studies aimed at developing biosurveillance systems for *H. halys* in freight containers, these studies were designed to detect pest prior to arrival in new areas. For example, Nixon et al. [27] explored the possibility of using volatile sampling from diapausing *H. halys* in freight as a means to detect *H. halys* prior to arrival. While in transit, it is likely that *H. halys* may not respond to baited pheromone traps, as pheromone-baited traps were not effective in capturing diapausing *H. halys* [28]. When combined with *H. halys* aggregation pheromone and MDT, clear sticky traps deployed in trees were used to successfully trap adults and nymphs in six European countries and Maryland, USA during the peak *H. halys* season [29]. However, it was unknown how those captures related to information that could have been generated using standard black pyramid traps, and whether captures were reliable over the course of the growing season. In our study, we targeted foraging *H. halys* populations, and we were able to demonstrate that a simpler, easy-to-use, clear sticky trap deployed on a wooden post and baited with pheromonal stimuli provided reliable captures of *H. halys* adults and nymphs season-long across different relative population densities with practical implications for agricultural and biosurveillance programs.

Comparing captures between the two trap designs across different population densities enabled us to distinguish their sensitivity. The standard pyramid trap captured relatively higher *H. halys* adults and nymphs than clear sticky traps; however, the difference between captures of the two traps was more obvious at moderate and high relative population density sites. This implies that in low relative density areas where *H. halys* is not well established or early in the season when *H. halys* populations are low, clear sticky traps are comparably sensitive to the pyramid traps for capturing adults and nymphs. As a result, clear sticky traps can be used for *H. halys* biosurveillance programs where the ability to detect nymphal and adult *H. halys* at low relative population densities is vital in delimiting incipient reproducing populations. In the case of the invasive emerald ash borer, *Agrilus planipennis* Fairmaire (Coleoptera: Buprestidae), using the lure-baited purple prism traps was found to be the most sensitive at capturing adults at low population densities [30] while tree branch removal was done to detect larval populations [31]. Recently, Valentin et al. [32] developed a rapid, sensitive approach for detecting *H. halys* presence based on the environmental DNA they leave behind while foraging. This approach has proven effective even when other trapping tools, particularly blacklight traps, did not necessarily detect their presence at low relative population densities. This approach offers another sensitive method for detecting *H. halys* presence, but it does not yet provide information regarding relative densities or differentiate between adult and nymphal lifestages, i.e., presence of reproductive populations. However, pheromone-baited sticky traps can provide this biological information concurrently. 

For monitoring *H. halys* populations in agricultural areas, the goal is not to capture as many individuals as possible, but rather to reliably monitor relative population densities across specified timeframes within or near affected crops with the least amount of time and effort; and relating population density changes to management. This trap-generated biological information can then assist in decision-making regarding the need for and timing of insecticidal applications. *Halyomorpha halys* captures in standard baited pyramid traps have been used as a guide for management in apple [23], suggesting that the same approach can be retooled for clear sticky traps. While captures were significantly higher in pyramid traps than on sticky traps at locations with moderate and high relative population densities, there were also significant positive correlations between captures of these two traps types with comparable trends of season-long captures for both adults and nymphs, demonstrating that the sticky traps could be used to reliably monitor *H. halys* presence and relative abundance in and near affected crops.

Aside from capture reliability, ease of deployment and maintenance are important considerations for developing a trap for agricultural monitoring and biosurveillance programs to reduce labor, time and cost requirements. In the case of *H. halys*, development of simplified trap designs using tree canopy-deployed traps such as funnel traps, small pyramid traps, delta traps, yellow sticky, and clear sticky traps have been tested [14,17,18,29], but, in general, sensitivity decreased significantly for nymphal captures in particular [17,18]. This decrease in captures of nymphs, can be attributed to their positively gravitactic behavior [33] which conflicts the fact that canopy-deployed traps are often hung from limbs, thereby requiring nymphs to walk ‘down’ rather than ’up’ to become captured. Moreover, for canopy-deployed traps, it is challenging to standardize the location considering that host species, canopy size and architecture may affect trap captures. Moreover, when using the *H. halys* aggregation pheromone and pheromone synergist as the stimuli source, the area of arrestment and retention capacity [34,35] of lure semiochemicals may affect capture rates especially when deployed within a tree as *H. halys* can aggregate on nearby limbs of the tree surrounding the baited trap (reviewed in 4). Hence, deploying the clear sticky trap atop a ground-deployed wooden stake offers a simple and standardized protocol for trap deployment that is also behaviorally compatible with the species.

Target specificity is an important consideration in trap development as well, as this minimizes labor and time required to sort through the traps and recover the target organism, and to help conserve beneficial nontarget species. Yellow sticky cards are commonly used to monitor presence and abundance of phytophagous insect pests and their respective natural enemies [36,37] due to its super-normal visual stimulus [25]. In our study, the effectiveness of the yellow sticky cards and clear sticky cards were comparable in capturing *H. halys* nymphs and adults, but yellow sticky cards attracted and retained more nontarget organisms than clear sticky cards. Although, we did not compare the nontarget captures in pyramid traps, we did observe different nontarget organisms inside the jar tops including predators, parasitic wasps and others (snails and tree frogs). Moreover, pyramid traps and sticky traps do require a certain amount of servicing. For the pyramid traps, the jar tops need to be cleaned including the funnels that serve as entry points, while for the sticky traps, the cards need to be replaced and/or the captured insects need to be removed. Rice et al. [18] discussed the costs and labor requirement associated with the pyramid traps.

The discovery of the two-component *H. halys* aggregation pheromone [20] and pheromone synergist, MDT [21] has paved the way for the advancement of monitoring tactics for this pest. Based on our two-year lure comparison studies, lures manufactured by Trécé resulted in greater captures in traps compared with lures manufactured by AgBio. Strong positive correlations between captures in pyramid and sticky traps baited with Trécé and AgoBio lures were observed consistently at all relative population densities; and captures in sticky traps baited with low and high loading rates of both lure types were correlated at very low and low relative population densities. Factors such as dispenser type, release rates, manufacturing process, and/or additives for chemical stability used by the two commercial lures may explain these differences. For example, traps baited with newly deployed MDT lures using an early formulation by another company, captured significantly more *H. halys* than lures that were three weeks or older likely because of rapid release of compounds after deployment [15]. Such differences in release rate for the two lure types evaluated may explain the differences we see here. On the other hand, purity of pheromonal components of *H. halys* was likely less important, as Leskey et al. [38] demonstrated nonpheromonal stereoisomers of 10,11-epoxy-1-bisabolen-3-ol were sufficiently similar to the true pheromonal stereoisomers of the *H. halys* pheromone. Moreover, a recent study has found that *H. halys* captures improved with the addition of ethyl (2*E*,4*E*,6*Z*)-decatrienoate (EDT) [39] suggesting that adding this attractant to the MDT and pheromone mix may further enhance trap sensitivity, although the added production cost should also be taken into consideration. While providing additional pheromonal components may enhance captures, Morrison et al. [40] found that the addition of host plant volatiles to pheromone and synergist lures did not enhance the captures of *H. halys* in baited traps.

In summary, we were able to provide evidence that using the clear sticky trap baited with pheromonal stimuli is a reliable, simpler and more practical alternative to the current standard pyramid trap for monitoring *H. halys* populations. To further substantiate the results from this study and help determine their applicability for biosurveillance programs, it may be useful to conduct a similar comparison between the standard pyramid trap and the clear sticky trap deployed across a larger geographical area with varying climatic conditions.

## Figures and Tables

**Table 1:** GPS coordinates of the study sites and the corresponding trapping period for the 2016 and 2017 biosurveillance studies for *H. halys*. The relative population density classification for each site is indicated in parenthesis.

Locations	GPS Coordinates	Trapping Period (Relative Population Density)	
**West Virginia**		**2016 Study**	**2017 Study**
Jefferson	39°22′52.41” N 77°52′49.81” W	23 May–3 October (M)	22 May–16 October (L)
Burns	39°14′49.30” N 77°55′5.59” W	23 May–3 October (M)	-
USGS *	39°21′16.89” N 77°54′39.89” W	23 May–3 October (H)	22 May–16 October (L)
Link *	39°20′31.12” N 77°53′45.25” W	23 May–3 October (M)	-
Tabb	39°20′41.52” N 77°55′27.78” W	23 May–3 October (M)	-
Twin Ridge *	39°22′42.59” N 77°50′42.81” W	23 May–3 October (H)	22 May–16 October (L)
**Virginia**			
Eastern Shore	37°35′23.44′′ N 75°49′11.82′′ W	31 May–3 October (L)	7 Jun–5 September (VL)
	37°38′21.18′′ N 77°58′36.93′′ W	6 June–3 October (M)	-
Goochland 2	37°41′49.65′′ N 78°01′8.89′′ W	6 June–3 October(L)	-
Goochland 3	37°40′45.97′′ N 77°53′32.29′′ W	6 June–3 October (M)	-
VA Site 1	37°12′20.49′′ N 80°33′53.60′′ W	31 May–26 September (M)	17 May–13 September (VL)
VA Site 2	37°11′28.07′′ N 80°34′42.73′′ W	31 May–26 September (M)	17 May–13 September (VL)

* Indicates sites with yellow sticky cards baited with Trécé low rate lures in comparison with clear sticky and pyramid traps from the week of 26 June to 3 October 2016. Population Density: “VL”: Very Low, “L”: Low; “M”: Moderate, “H”: High.

**Table 2:** Pearson correlation coefficients (α = 0.05) between captures of *H. halys* in pyramid traps compared with clear sticky cards under low, moderate, and high relative population densities in 2016.

Lure Type & Population Density	Adults			Nymphs		
	*r*	*df*	*p*	*r*	*df*	*p*
Trécé Low						
Low	0.817	37	0.0001	0.832	37	0.0001
Moderate	0.739	152	0.0001	0.544	152	0.0001
High	0.803	40	0.0001	0.559	40	0.0002
Trécé High						
Low	0.782	37	0.0001	0.661	37	0.0001
Moderate	0.636	152	0.0001	0.695	152	0.0001
High	0.801	40	0.0001	0.615	40	0.0001
AgBio Low						
Low	0.822	25	0.0001	0.433	25	0.0305
Moderate	0.759	130	0.0001	0.467	130	0.0001
High	0.752	36	0.0001	0.114	36	0.507
AgBio High						
Low	0.820	25	0.0001	0.741	25	0.0001
Moderate	0.719	130	0.0001	0.451	130	0.0001
High	0.726	36	0.0001	0.287	36	0.090

**Table 3:** Pearson correlation coefficients (α = 0.05) between captures of *H. halys* using low and high loading rates of Trécé and AgBio lures on clear sticky traps in 2016 and 2017.

Lure Type & Population Density	2016						Lure Type & Population Density	2017					
	Adults			Nymphs				Adults			Nymphs		
	*r*	*df*		*r*	*df*			*r*	*df*		*r*	*df*	*p*
Trécé lures							Trécé lures						
Low	0.812	37	0.0001	0.696	37	0.0001	Very Low	0.598	58	0.0001	0.825	50	0.0001
Moderate	0.87	152	0.0001	0.736	152	0.0001	Low	0.889	66	0.0001	0.788	66	0.0001
High	0.889	40	0.0001	0.647	40	0.0001	-	-	-	-	-	-	-
AgBio lures													
Low	0.618	25	0.001	0.741	25	0.0001	Very Low	0.386	50	0.0056	0.68	50	0.0001
Moderate	0.787	130	0.0001	0.719	130	0.0001	Low	0.855	66	0.0001	0.553	66	0.0001
High	0.931	36	0.0001	0.147	36	0.3934	-	-	-	-	-	-	-

## Supplementary Materials

The following are available online at http://www.mdpi.com/2075-4450/9/3/82/s1, Figure S1: Seasonal captures of *H. halys* adults at each field site from 6 June to 26 September 2016. Figure S2: Captures of *H. halys* adults at each field site from 5 June to 4 September 2017.
