# Supplementary material for: Monitoring and Biosurveillance Tools for the Brown Marmorated Stink Bug, Halyomorpha halys (Stål) (Hemiptera: Pentatomidae)
Source: Insects. 2018 Jul 8;9(3):82. doi: 10.3390/insects9030082 (PMC6163172; doi:10.3390/insects9030082)
Supplement: Supplementary file 1 [file insects-09-00082-s001.pdf]

Supplemental Materials

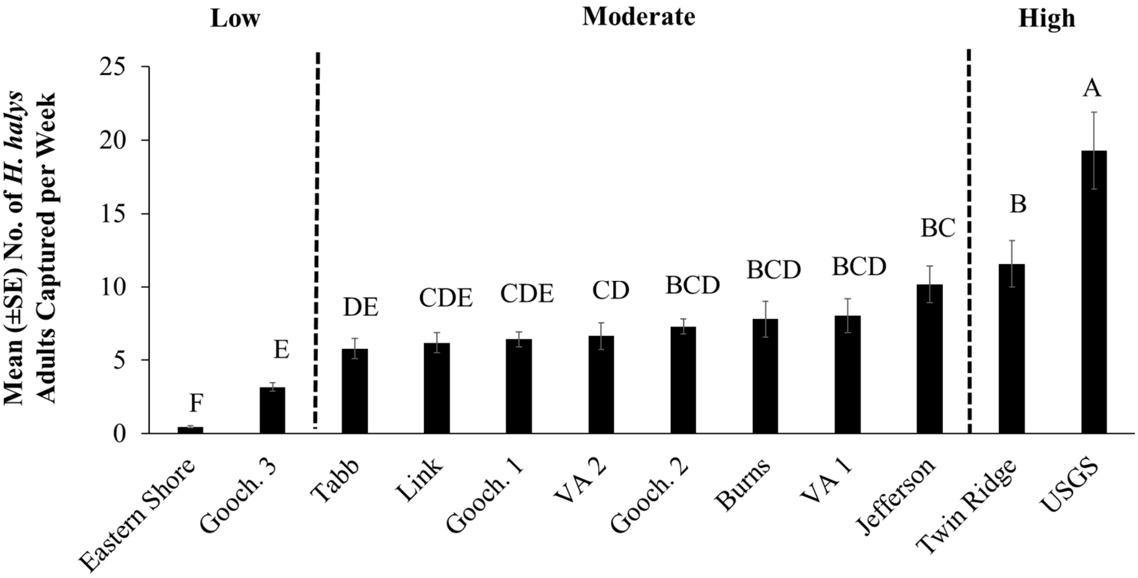

Figure S1. Seasonal captures of *H. halys* adults at each field site from 6 June to 26 September 2016. Bars with shared letters are not significantly different from each other at $\alpha = 0.05$ (χ^2 mean contrast with Bonferroni correction). Sites were classified according to relative population densities found at each site and grouping was based on the results of the post-hoc test. Data were pooled across all trap and lure treatments.

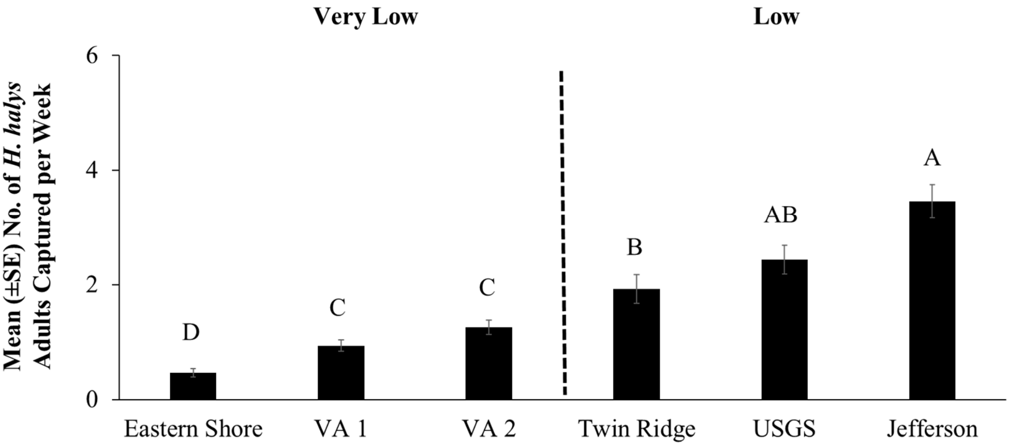

Figure S2. Captures of *H. halys* adults at each field site from 5 June to 4 September 2017. Bars with shared letters are not significantly different from each other at $\alpha = 0.05$ (χ^2 mean contrast with Bonferroni correction). Sites were classified according to relative population density found at each site and grouping was based on the results of the post-hoc test. Data were pooled across all treatments.
